# Red Vetchling (*Lathyrus cicera* L.), a Promising Crop for the Sustainable Replacement of Soybean Meal and Reducing the Carbon Footprint of European Aquafeeds

**DOI:** 10.3390/ani13203178

**Published:** 2023-10-11

**Authors:** Francisco Javier Toledo-Solís, Amin Mokhles Abadi Farahani, Sara Yagüe, Inmaculada Mateos-Aparicio, Valentín Pérez, Ana María Larrán, Francisco Javier Moyano, Ignacio Fernández

**Affiliations:** 1 Department of Biology and Geology, University of Almeria, 04120 Almeria, Spain; fjmoyano@ual.es; 2 Centro de Investigaciones Costeras-ICBiol-UNICACH, Calle Juan José Calzada s/n, Tonalá 30500, Chiapas, Mexico; 3 Department of Natural Resources (Fisheries Division), Isfahan University of Technology (IUT), Isfahan 8415683111, Iran; amin.mokhles@na.iut.ac.ir; 4 Centro Oceanográfico de Vigo, Instituto Español de Oceanografía, (IEO), CSIC, Subida a Radio Faro 50, 36390 Vigo, Spain; 5 Centro de Investigación en Acuacultura, Instituto Tecnológico Agrario de Castilla y León (ITACyL), Ctra. Arévalo, Zamarramala, 40196 Segovia, Spain; sarayague@hotmail.com (S.Y.); largaran@itacyl.es (A.M.L.); 6 Facultad de Farmacia, Universidad Complutense de Madrid, 28040 Madrid, Spain; inmateos@ucm.es; 7 Campus Vegazana s/n, Universidad de León, 24071 León, Spain

**Keywords:** local crops, antinutritional factors, non-starch polysaccharides, rainbow trout

## Abstract

**Simple Summary:**

The sustainability of aquaculture’s growth is a worldwide challenge in the coming decades. Among the different approaches considered (e.g., implementation of recirculation systems, zero waste, etc.), reducing the use of raw materials imported from third countries might help to lower the carbon footprint of European aquaculture. Soybean meal (SBM) is one of the most widely used alternative raw materials to replace fishmeal, but is largely imported from Argentina, Brazil, and/or USA. Red vetchling (*Lathyrus cicera*) is a crop locally produced in Europe that might reduce the European dependency on SBM imports. Here, the replacement of SBM with red vetchling in diets for rainbow trout (*Oncorhynchus mykiss*) juveniles has been evaluated in different percentages (from 0 to 100%). Only fish fed a diet where SBM has been replaced at 100% showed lower growth, an altered amino acid profile in fish fillet, and some histopathological signs in the liver (greater % of pyknotic nuclei). Also, although temporally, glucose and triglycerides blood plasma levels were reduced, normal values were found at 24 h after feeding. Since vetchling meal can partially replace SBM without a negative effect on fish growth or physiology, its use might allow the reduction of SBM imports and the carbon footprint of European aquaculture.

**Abstract:**

In fish diets, soybean meal (SBM) is still positioned as the most widely used alternative to replace fishmeal. Red vetchling (*Lathyrus cicera*), a crop locally produced in Europe, is here evaluated as a substitute for SBM. Rainbow trout (*Oncorhynchus mykiss*) juveniles (10.34 ± 0.04 g) were fed for 90 days. Six experimental diets replacing the SBM content at 0, 8, 16, 33, 66, and 100% (Named Control, T8, T16, T33, T66, and T100) were tested. Growth performance and fish fillet amino acid composition were only significantly reduced in fish fed the T100 diet. Histopathological analysis showed that no major alterations were observed in the intestine, while T100 fish had a higher density of pyknotic nuclei in the hepatocytes than the Control, but similar hepatocyte surface coverage. Finally, postprandial levels of glucose and triglycerides in blood plasma decreased when red vetchling content was increased, but was only not fully restored after 24 h in the case of glucose in T66 and T100 fish. According to these results, red vetchling meal can replace up to 66% of the SBM without a negative effect on fish growth or physiology, representing a good alternative raw material for reducing European aquaculture’s dependency on SBM imports and the carbon footprint of aquafeeds.

## 1. Introduction

In recent decades, the rapid development of aquaculture has made a great contribution to coping with the increasing demand of animal protein for human consumption. Although fish meal (FM) is the most suitable protein source for many farmed fish, dwindling marine resources, the sharp increase of FM demand and its prices have forced the identification and use of alternative protein sources [1,2]. Nowadays, soybean meal (SBM) is still positioned as the most widely used alternative vegetable meals to replace FM [3]. This is due to its balanced amino acid profile, constant and high protein content, worldwide availability, and reasonable price [4]. Nevertheless, the use of SBM in diets for high trophic level (“carnivorous”) fish species is limited by the presence of antinutritional factors (ANFs) such as protease inhibitors, lectins, phytic acid, saponins, phytoestrogens, antivitamins, and allergens. Indeed, ANFs are known to reduce palatability, efficient utilization of feed nutrients, alter nutrient balances of the diets, inhibit growth, induce intestinal dysfunction (at cellular level), alter redox balance and gut microbiota, reduce immunocompetence, and/or induce pancreatic hypertrophy, hypoglycemia, or liver damage [5,6]. Furthermore, the SBM market functions on a global scale, and large volumes are only available from North and South America, which are the major producing regions [7]. In 2023, the EU only produced 2 million of the 28–29 million tons consumed. Therefore, for less dependent and more sustainable European aquaculture, the implementation of locally produced legumes in animal feeds is needed [7,8]. This will help to reduce the carbon footprint of European aquafeeds and its production costs.

Red vetchling (*Lathyrus cicera* L.) is most likely one of the first crops domesticated in Europe, and has considerable potential for producing more than 8 tons of forage dry matter and around 3.6 tons of dry grain per hectare, with a dry matter crude protein content surpassing 250 g per kg [9]. However, the use of *Lathyrus* spp. for human consumption is well known to induce paralysis due to its content in neurotoxin 3-(-N-oxalyl)-L-2,3-diamino propionic acid (ODAP; [10]). Nevertheless, red vetchling can be safely included at up to 40, 30, and/or 70% in the diet of poultry, pigs, and sheep, respectively [11]. However, as in the case of other plant-derived raw materials, the presence of other ANFs might also limit its implementation in fish diets [6,12,13]. This might be the particular case of the high content in carbohydrates and/or the presence of different non-starch polysaccharides (NSPs) in red vetchling [11]. In fact, since fish and other monogastric animals do not have enzymes such as β-glucanases or β-xylanases to digest them [14], the content of NSPs in red vetchling could be a problem.

Here, the use of red vetchling meal in the diet of rainbow trout (*Oncorhynchus mykiss*), one of the most largely farmed fish species worldwide (>800,000 t y^−1^; [15]), and a glucose intolerant model species [16] was explored. The present study aims to identify the maximum level of SBM replacement with locally produced red vetchling meal in order to achieve a more sustainable and independent European aquaculture by reducing the carbon footprint and the costs of aquafeeds.

## 2. Materials and Methods

### 2.1. Ethical Statement

All experiments were conducted following the ARRIVE (Animal Research: Reporting of In Vivo Experiments) guidelines [17], according to 2010/63/EU of the European Parliament and Council, guideline 86/609/EU of the European Union Council and Spanish legislation (RD53/2013), and were previously approved by the Bioethical Committee of ITACyL (approval number: 2018/31/CEEA).

### 2.2. Experimental Design, Diets and Sampling

Six experimental isonitrogenous and isoenergetic diets (Table 1) were formulated and produced at the Experimental Feeds Unit of the University of Almeria (Almeria, Spain). Experimental diets differed in the percentages of the substitution of SBM by red vetchling meal: 0, 8, 16, 33, 66, and 100% (named Control, T8, T16, T33, T66, and T100, respectively). Red vetchling seeds, provided by a local producer, were only ground (to 500 µm) before being included in the mixture.

A total of 360 rainbow trout juveniles with a mean wet weight of 10.34 ± 0.04 g and an initial furcal length of 9.45 ± 0.02 cm g were randomly distributed (20 individuals/tank and 3 tanks per diet) in 18 cylindroconical 500 L tanks, connected to a recirculation system. The dose–response trial was performed for 90 days under controlled parameters of temperature (15 °C), dissolved oxygen (>7 mg/L), and a light:dark photoperiod of 12:12 h. Ammonium and nitrite concentrations were monitored daily to keep them below toxic values. Animals were fed daily in the morning (between 08:00–09:00 h) until apparent satiety, using a maximum of 3% of the biomass as a daily feed intake. Every 21 days, the growth of the fish was monitored and the feeding ration and pellet size adjusted. Different feed pellets (2, 3.5, and 5 mm) were used throughout the trial.

At the end of the trial, the fish were sampled for growth performance, proximate and amino acid composition of the red vetchling meal and fish fillet, histopatho-morphological analysis of the liver and proximal intestine, as well as for blood biochemistry.

### 2.3. Proximate Composition

Moisture and protein content in the different matrices were determined according to Association of Official Agricultural Chemists (AOAC) procedures [18] and the Official Journal of the European Union [19]. Moisture was calculated by drying samples at 105 °C for 24 h until constant weight. Protein content was analyzed using the Kjeldahl method (N × 6.25), fat using dichloromethane extraction (Soxhlet), and ash content by heating the residue from the moisture determination in a muffle furnace at 550 °C for 24 h. All parameters were expressed in percentage in relation to dry matter. Analyses were conducted in triplicates in the case of red vetchling meal. For fish fillet, muscle samples from 3 fish per tank (3 tanks per experimental group) were analyzed.

### 2.4. Amino Acid Profile

The amino acid analysis profile of red vetchling meal was performed in triplicates using 20 mg of meal. For fish fillet, muscle samples from 3 fish per tank (3 tanks per experimental group) were analyzed. After hydrolysis with 1 mL of 6 N HCl for 24 h at 110 °C, samples were neutralized with NaOH 6.5 N and were diluted ten times with loading buffer pH 2.2 (80-2037-67, Biochrom, Cambridge, UK). The determination was performed by ion-exchange liquid chromatography and post-column continuous reaction with ninhydrin (Biochrom 30+, Cambridge, UK) to provide qualitative and quantitative compositional analysis. Norleucine was used as the internal standard.

### 2.5. Profile of Non-Starch Polysaccharide

Dietary fiber was determined according to the AOAC enzymatic–gravimetric method 991.43. The gravimetric residues were treated with H_2_SO_4_ 12 M at 35 °C for 30 min, followed by H_2_SO_4_ 2 M at 100 °C for 1 h. The released neutral sugars from the NSPs were transformed into alditol acetates with acetic anhydride in the presence of 1-methylimidazol and were quantified by GLC using β-D-allose (Fluka) as the internal standard in a Perkin-Elmer Autosystem Chromatograph (Waltham, MA, USA) equipped with a hydrogen flame ionization detector. The column used was an SP-2330 (30 m long, 0.25 mm i.d., and 0.25 μm film thickness) and nitrogen served as the carrier gas. Injector and detector temperatures were 275 °C and oven temperature 235 °C. Uronic acid content was determined according to the colorimetric method of 3,5-dimethylphenol previously miniaturized and adapted to a microplate reader (Synergy^TM^ HTX Multi-Mode, BioTek, Winooski, VT, USA), using galacturonic acid monohydrate (Merck) as standard. Total NSP was calculated as the sum of both neutral and acid sugars, and results were expressed as g/100 g of raw dry material. Analyses were conducted on three independent samples of red vetchling meal.

### 2.6. Growth Performance

Fish were slightly anesthetized with MS-222 (0.08 g/L), before recording furcal length with a graduated ichthyometer (0.1 mm) and the wet weight with a GRAM S3R-6KD balance (0.1 g). All the fish from each tank (with 3 tanks per experimental group) were measured and weighed. Feed consumption in each tank and mortality were recorded daily. At the end of the experiment, weight gain (WG), specific growth rate (SGR), feed conversion ratio (FCR), condition factor (CF), and viscerosomatic and hepatosomatic indices were calculated according to what was described in [20].

### 2.7. Digestibility Assay

For the apparent digestibility analysis, feces were collected 24 h after feeding during the last two weeks of the trial. The feces of each experimental unit were stored at −80 °C until their respective analysis. The apparent digestibility of the protein was determined using acid-insoluble ash as a marker in feces [21], and was calculated as follows: ADCprot (apparent digestibility coefficient of protein) = 100 − [(marker in diet/marker in feces) × (% protein in feces/% protein in diet) × 100]. Apparent digestibility was analyzed in three technical replicates from each biological unit (tank).

### 2.8. Postprandial Analysis

After the 90 days post feeding trial, fish were also sampled at 3, 6, and 24 h after feeding, using a total of 9 fish per treatment (3 fish from each tank). Blood was taken from the caudal vein using 1 mL plastic syringes coated with lithium heparin as an anticoagulant, and was transferred to 1 mL tubes with lithium heparin (MiniCollect^®^, San Sebastián de los Reyes, Spain). Plasma was obtained by centrifugation at 5000 rpm for 20 min at 4 °C and stored at −80 °C until analysis.

The determination of glucose and triglycerides in plasma was performed with glucose-RT (BSIS17_E) and triglycerides (BSIS31_E) colorimetric assay kits according to the manufacturer’s instructions (Spinreact^®^, Girona, Spain). Absorbance was measured in 96-well microplates using a microplate reader (ELx800TM; BioTek Instruments, Inc., Winooski, VT, USA).

### 2.9. Histopathological Analysis

At the end of the experiment, dissected liver and proximal intestine tissues of rainbow trout were fixed by immersion in 4% buffered paraformaldehyde (pH 7.4) for 24 h at room temperature. The fixed samples were dehydrated by transferring them in a sequential series of graded alcohol solutions (25%, 50%, 75%, and 100%). They were then embedded in paraffin blocks, sectioned (3–5 µm), and stained with hematoxylin-eosin and Alcian blue (AB, pH = 2.5)-periodic acid-Schiff (PAS) solutions to characterize liver and intestine histomorphology, as well as to identify and quantify the goblet cell’s density in intestinal sections. All procedures were performed as described by [22,23].

The mounted sections were analyzed using a Leica digital microscope (model DM 4000B; Leica, Wetzlar, Germany). The following parameters were assessed at the proximal intestine of 3 fish per experimental tank: the height of villi and enterocytes, submucosa, muscular, and serosa layer width, and goblet cell density. In the liver, hepatocyte ballooning, vacuolar degeneration, number of pyknotic nuclei per segment, capillary hyperemia, vasculature dilation, and hepatocyte surface coverage (% of surface covered by hepatocyte cytoplasm compared to that covered by vacuoles) were evaluated. To measure hepatocyte’s surface coverage, the white area of the liver was analyzed with image J (version 1.52) software. For any evaluated parameter, at least 3 measurements per section were made.

### 2.10. Statistical Analysis

Results are presented as mean values ± standard deviations from 3 biological replicates (tanks) for each experimental group. All data were previously checked for normality (Kolmogorov–Smirnov test) and homoscedasticity of variance (Bartlett’s test). The results were compared using a one-way ANOVA followed by Tukey’s test. The significance level was set at *p* < 0.05, and all statistical analyses were performed using GraphPad Prism 5.0 (GraphPad Software, Inc., San Diego, CA, USA).

## 3. Results

### 3.1. Nutritional Characteristics of Red Vetchling and the Experimental Diets

Red vetchling nutritional composition is shown in Table 2. Crude protein content in red vetchling seeds accounted for 23.82 ± 0.47%, and crude fat for 2.03 ± 0.06%. Moisture represented 3.62 ± 0.22%, ash 3.78 ± 0.07%, and Nfe (Nitrogen-free extract) 70.37 ± 0.39%. Red vetchling seeds include all the essential and non-essential amino acids, the lowest contents being found in histidine, methionine, and cysteine (0.38 ± 0.02, 0.24 ± 0.01, and 0.15 ± 0.01 g/100 g, respectively), and the highest contents in glutamic acid, aspartic acid, proline, arginine, and lysine (4.19 ± 0.12, 2.75 ± 0.06, 1.89 ± 0.20, 1.88 ± 0.12, and 1.75 ± 0.10 g/100 g, respectively) (Table 2).

The main cell wall monomers found in red vetchling (Table 3) were glucose (3.82 ± 0.36 mg/g), arabinose (2.79 ± 0.33 mg/g), and galacturonic acid (2.28 ± 0.46 mg/g). Less abundant monomers were mannose (0.26 ± 0.05 mg/g) and galactose (0.38 ± 0.05 mg/g). No rhamnose or fucose were detected. This composition indicates that the main non-starch polysaccharides (NSPs) would be cellulose, arabinans, galactomannans, and homogalacturonans. The highest proportion of these monomers was found in the insoluble fraction.

The amino acid profile of the experimental diets (Table 4) shows quite a balanced profile for all the essential amino acids. Only a significantly lower content in leucine was found in T16, T33, and T66 diets (ranging from 2.63 ± 0.05 to 2.64 ± 0.05 g/100 g) when compared to the Control diet (2.89 ± 0.07 g/100 g; ANOVA, *p* < 0.05). Regarding the non-essential amino acids, alanine, aspartic acid, glutamic acid, and glycine showed differences among the different experimental diets. The amount of alanine was only significantly lower in the T100 diet (1.86 ± 0.02 g/100 g) when compared with the Control (2.07 ± 0.01 g/100 g) and T8 (2.07 ± 0.00 g/100 g; ANOVA, *p* < 0.05) diets. The amount of aspartic acid also significantly decreased when the SBM replacement by red vetchling was increased. The highest content was observed in the Control (3.40 ± 0.06 g/100 g) and T8 (3.37 ± 0.03 g/100 g) diets, while the lowest was found in the T100 diet (2.69 ± 0.04 g/100 g; ANOVA, *p* < 0.05). Aspartic acid content in T16, T33, and T66 was also significantly reduced when compared to the Control and T8 diets. The highest amount of glutamic acid was reported in the T100 (9.20 ± 0.08 g/100 g) diet, whereas in the case of glycine, the highest content was found in the T33 (2.02 ± 0.06) diet.

### 3.2. Rainbow Trout Growth Performance When Fed Experimental Diets

Growth performance indicators are compiled in Table 5. After a 90-day dose–response trial, only fish fed the T100 diet showed significantly lower final body weight than those fed with the Control diet (100.5 ± 5.8 vs. 150.7 ± 2.3 g, respectively; ANOVA, *p* < 0.05). Similar differences were only reported between these two experimental groups regarding the final furcal length (20.2 ± 0.4 vs. 22.7 ± 0.2 cm, respectively; ANOVA, *p* < 0.05), weight gain (871.9 ± 60.1 vs. 1357.8 ± 18.2 g, respectively; ANOVA, *p* < 0.05), specific growth rate (2.53 ± 0.07 vs. 2.98 ± 0.01%/day, respectively; ANOVA, *p* < 0.05), feed conversion rate (1.00 ± 0.04 vs. 0.79 ± 0.01, respectively; ANOVA, *p* < 0.05), and condition factor (1.23 ± 0.01 vs. 1.29 ± 0.02, respectively; ANOVA, *p* < 0.05). While no significant differences were found in the viscerosomatic index (with values ranging from 10.19 ± 2.62% to 12.00 ± 0.90%), a significantly lower hepatosomatic index in fish fed the T66 diet was found when compared to that of the Control fish (1.13 ± 0.02% vs. 1.42 ± 0.12%, respectively; ANOVA, *p* < 0.05), with fish fed T8, T16, T33, and T100 showing intermediate values.

Apparent protein digestibility was significantly reduced in the dietary group with the highest SBM replacement with red vetchling (T100) when compared to that of the Control group (90.03 ± 0.84% vs. 92.89 ± 0.18%, respectively; ANOVA, *p* < 0.05), while it was similar or even higher for the other diets containing the red vetchling replacement. Fish fillet proximate composition also varied depending on SBM replacement with red vetchling. Fish fillet moisture was highest in fish fed the T33 diet (2.41 ± 0.88%), although this was not significantly different when compared with that of fish fed the Control diet (1.74 ± 0.15%; ANOVA, *p* > 0.05), and lowest in fish fed the T100 diet (0.68 ± 0.28%; ANOVA, *p* < 0.05). The amount of crude protein decreased when SBM replacement increased, the lowest values being found in fish fed the T66 and T100 diets (with values ranging from 11.06 ± 0.31% to 13.06 ± 0.04%) and the highest in fish fed the Control and T8 diets (15.56 ± 0.21% and 16.31 ± 0.54%, respectively; ANOVA, *p* < 0.05). In contrast, ash content was only significantly reduced in fish fed the T66 and T100 diets (3.25 ± 0.15% and 2.66 ± 0.15%, respectively) when compared to that of the Control fish (4.69 ± 0.06%; ANOVA, *p* < 0.05).

### 3.3. Amino Acid Composition of Muscle

At the end of the 90-day experimental period, fish fillets from all the experimental groups showed an almost fully balanced amino acid profile (Table 6). No significant differences were observed among the essential amino acid, including arginine (ranging from 3.33 ± 0.24 to 4.06 ± 0.44 g/100 g), histidine (ranging from 2.13 ± 0.72 to 3.05 ± 0.50 g/100 g), isoleucine (ranging from 4.31 ± 0.32 to 4.68 ± 0.42 g/100 g), leucine (ranging from 7.22 ± 0.37 to 7.69 ± 0.57 g/100 g), lysine (ranging from 6.82 ± 0.59 to 8.08 ± 0.64 g/100 g), phenylalanine (ranging from 3.53 ± 0.19 to 4.12 ± 0.50 g/100 g), valine (ranging from 3.31 ± 0.17 to 3.55 ± 0.18 g/100 g), methionine (ranging from 2.63 ± 0.19 to 3.23 ± 0.54 g/100 g), and threonine (ranging from 2.16 ± 0.04 to 2.50 ± 0.22 g/100 g). Among the non-essential amino acids, alanine, aspartic acid, glutamic acid, glycine, serine, cysteine, and proline were not significantly altered in any experimental group (ANOVA, *p* > 0.05). The only exception was tyrosine, where the T100 group showed the highest content (2.89 ± 0.24 g/100 g).

### 3.4. Histopathological Analysis of Liver and Proximal Intestine

**Figure 1:**
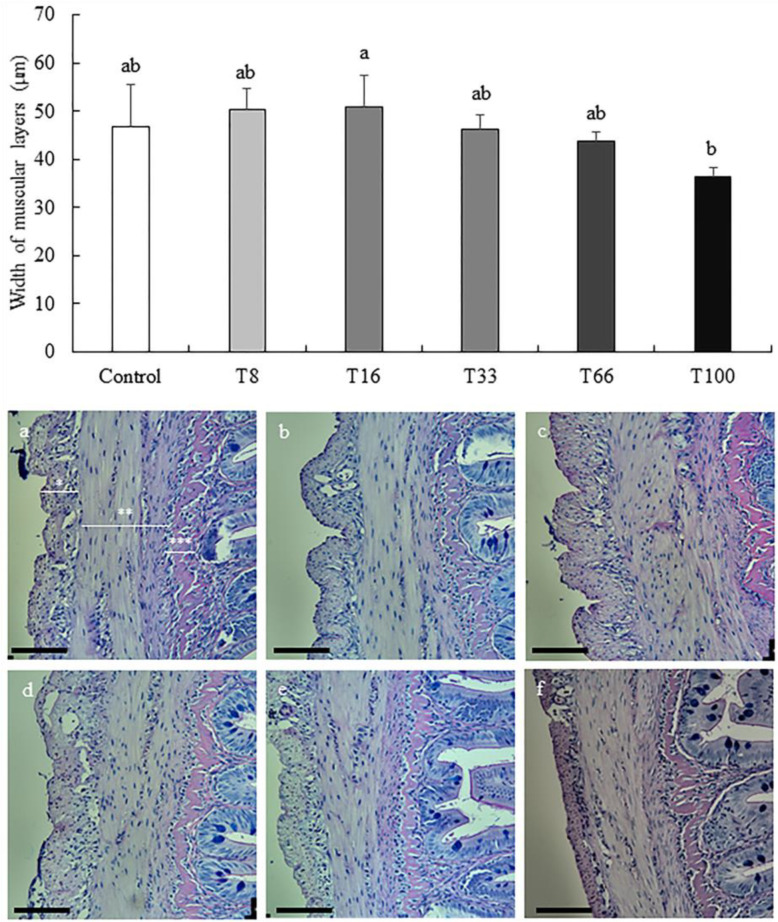
Width (mean ± SD) of muscular layers (µm) of proximal intestine in rainbow trout (*Oncorhynchus mykiss*) fed different diets containing increasing levels of soybean meal replacement by red vetchling (*Lathyrus cicera*) meal. Representative images of the proximal intestine in fish fed with Control (**a**), T8 (**b**), T16 (**c**), T33 (**d**), T66 (**e**), and T100 (**f**) diets. * Serosa layer, ** Muscular layers, *** Submucosa layer. Proximal intestine sections were stained with Alcian Blue and PAS. Images were recorded with a Leica digital camera (model DMC 5400; Leica, Wetzlar, Germany) connected to a Leica microscope (model DM 4000B; Leica, Wetzlar, Germany). Scale bare = 100 µm. Different letters at the top of each histogram denote significant differences (one-way ANOVA, *p* < 0.05; *n* = 3).

A thorough liver and proximal intestine histopathological analysis was conducted to assess the impact of SBM replacement with red vetchling in rainbow trout diets (Table 7). According to the histology of the proximal intestine, no significant differences were observed regarding submucosa layer width (ranging from 6.06 ± 1.86 to 8.82 ± 0.72 µm; ANOVA, *p* > 0.05) or serosa layer (ranging from 12.47 ± 1.80 to 16.54 ± 1.79 µm; ANOVA, *p* > 0.05), height of the villi (ranging from 338.31 ± 27.69 to 357.68 ± 35.12 µm; ANOVA, *p* > 0.05), and enterocytes (ranging from 14.46 ± 1.49 to 15.47 ± 0.26 µm; ANOVA, *p* > 0.05), or in goblet cells density (ranging from 50.33 ± 5.75 to 61.82 ± 18.04 cells/mm; ANOVA, *p* > 0.05). In contrast, muscular layer width was significantly greater in the T16 group (50.82 ± 6.76 µm) when compared with that of the T100 group (36.31 ± 2.04 µm; ANOVA, *p* < 0.05; Figure 1), with the other experimental groups showing intermediate values.

**Figure 2:**
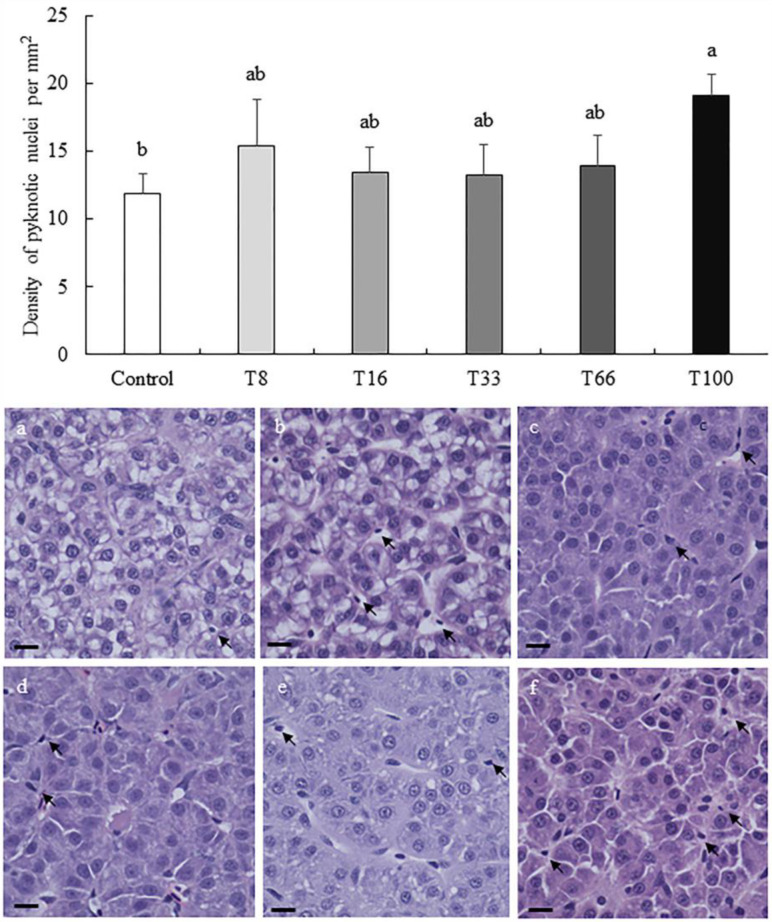
Density (mean ± SD) of pyknotic nuclei per mm^2^ in liver sections from rainbow trout (*Oncorhynchus mykiss*) fed different diets containing increasing levels of soybean meal replacement by red vetchling (*Lathyrus cicera*) meal. Representative images of the proximal intestine in fish fed with Control (**a**), T8 (**b**), T16 (**c**), T33 (**d**), T66 (**e**), and T100 (**f**) diets are shown. *Arrows*, pyknotic nuclei. Liver sections were stained with hematoxylin-eosin. Images were recorded with a Leica digital camera (model DMC 5400; Leica, Wetzlar, Germany) connected to a Leica microscope (model DM 4000B; Leica, Wetzlar, Germany), scale bar = 25 µm. Different letters at the top of each histogram denote significant differences (one-way ANOVA, *p* < 0.05; *n* = 3).

**Figure 3:**
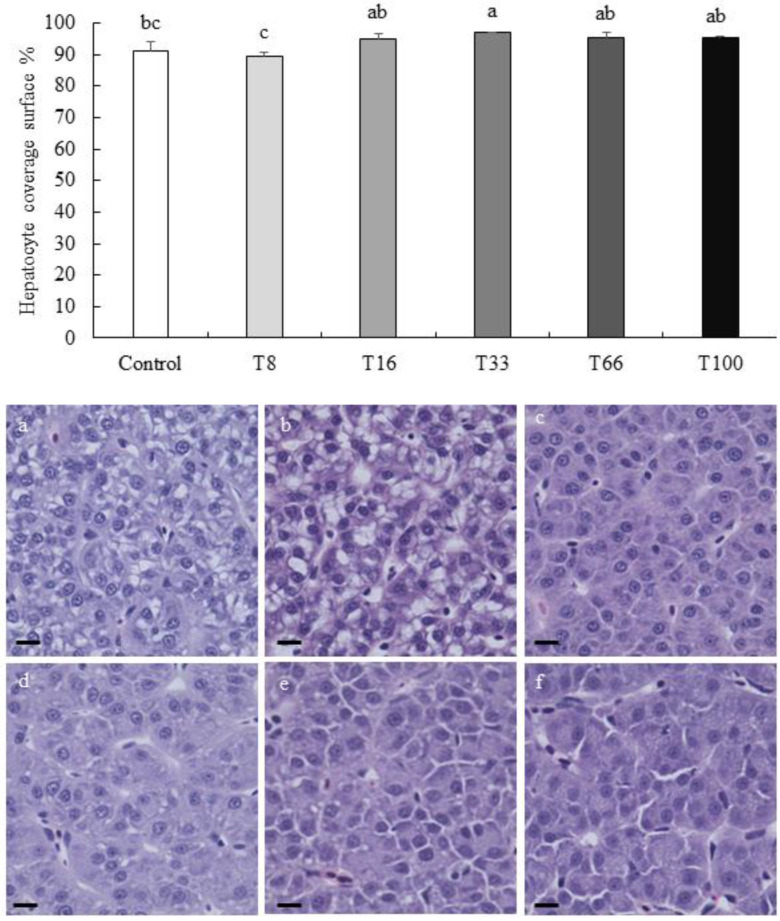
Hepatocyte surface coverage in percentage (mean ± SD) in liver sections from rainbow trout (*Oncorhynchus mykiss*) fed different diets containing increasing levels of soybean meal replacement by red vetchling (*Lathyrus cicera*) meal. Representative images of the liver in fish fed with Control (**a**), T8 (**b**), T16 (**c**), T33 (**d**), T66 (**e**), and T100 (**f**) diets are shown. Liver sections were stained with hematoxylin-eosin. Images were recorded with a Leica digital camera (model DMC 5400; Leica, Wetzlar, Germany) connected to a Leica digital microscope (model DM 4000B; Leica, Wetzlar, Germany), scale bar = 25 µm. Different letters at the top of each histogram denote significant differences (one-way ANOVA, *p* < 0.05; *n* = 3).

In liver, the density of cells with a pyknotic nucleus increased with the dietary increase of red vetchling (Figure 2). However, this was only significantly higher in the T100 group when compared to the Control group (19.11 ± 1.57 vs. 11.90 ± 1.44 pyknotic nuclei per mm^2^; ANOVA, *p* < 0.05). Regarding hepatocyte surface coverage, the lowest coverage was found in liver from fish fed the T8 diet (89.39 ± 1.38%), and the highest in the T33 group (96.83 ± 1.92%; ANOVA, *p* < 0.05; Figure 3). No hepatocyte ballooning, vacuolar degeneration, capillary hyperemia, or vasculature dilation were detected among the different experimental groups.

### 3.5. Postprandial Plasma Levels of Glucose and Triglycerides

The blood plasma biochemistry results at the end of the study (90 days feeding period) are shown in Table 8. Glucose level in plasma at 3 h post-feeding was reduced when red vetchling dietary content increased, T66 and T100 showing the lowest levels (73.6 ± 10.0 and 73.8 ± 7.5 mg/dL, respectively) compared to those of the Control and T8 groups (100.4 ± 10.2 and 117.3 ± 12.1 mg/dL, respectively). At 6 h post-feeding, only fish fed the T100 diet still showed reduced glucose plasma levels when compared with the Control fish (62.5 ± 3.1 vs. 79.9 ± 2.4 mg/dL, respectively; ANOVA, *p* < 0.05). At 24 h post-feeding, glucose levels in T66 and T100 remained low (66.9 ± 7.2 and 67.8 ± 5.3 mg/dL, respectively) when compared to those of the Control fish (100.2 ± 13.2 mg/dL; ANOVA, *p* < 0.05).

Plasma levels of triglycerides from 3 to 24 h post-feeding followed a similar trend. At 3 h post-feeding, fish fed diets containing red vetchling showed significantly reduced plasma levels when compared to those of the Control fish (from 134.8 ± 24.7 to 229.7 ± 36.5 vs. 377.8 ± 48.0 mg/dL; ANOVA, *p* > 0.05). At 6 h post-feeding, blood plasma triglycerides levels were still reduced in fish from the T16–100 groups, but were fully restored and equal to those from the Control fish at 24 h post-feeding (ANOVA, *p* > 0.05).

## 4. Discussion

Red vetchling is a legume with low production cost that is highly adapted to environments with unfavorable conditions including drought or low temperatures, with enhanced resistance to powdery mildew, pseudomonas, crenate broomrape (*Orobanche crenata*), or rust [9,11]. The suitability of red vetchling as an alternative source of protein for fish diets was previously evaluated in vitro regarding its total buffering capacity, inhibition of alkaline protease activity, soluble protein, phytic phosphorus, total soluble phosphorus, and phenolic compounds content, and was compared to other potential vegetable protein sources [8]. Red vetchling was also reported to contain numerous bioactive compounds, mainly antioxidants (e.g., polyphenols and vitamins) [9]. Its protein content values are known to be lower than that of soybean and lupin (*Lupinus* spp.), but higher than that of green pea (*Pisum sativum*) and faba bean (*Vicia faba*), ranging between 22 and 31% [9]. In contrast to lupin, red vetchling has a low fat content (slightly less than 5% of lipids) but a high carbohydrate content (40–60%; reviewed in [11]). These values depend on the variety, season, and/or region [11,12,24,25]. Mineral content is also similar to other agriculturally important grain legumes [11]. Results of the proximate composition presented here are within the values reported by the literature.

As an alternative protein source, the amino acid profile of red vetchling seeds was quite balanced, and comparable to that reported for many grain legumes, being deficient in the sulphur-containing amino acids (methionine and cystine) but rich in lysine [11,25]. However, the experimental diets formulated here only showed slight differences in leucine. Further differences were also seen in non-essential amino acids alanine, aspartic acid, glutamic acid, and glycine but their levels were always higher than those required for rainbow trout [26]. In fact, when analyzing the amino acid profile of fish fed the different experimental diets, only minor differences were found for tyrosine.

The presence of different ANFs might be the most limiting factor for using red vetchling for human and animal nutrition [9,12,27]. On the one hand, different reports have indicated the presence of ODAP in *L. cicera* and *L. sativus* [28], a neurotoxic compound inducing lathyrism in a diverse set of livestock species (reviewed in [11]). Nevertheless, new cultivars with an outstanding reduced ODAP content (1.5 to 0.01%) and/or processing measures, such as incorporating zinc sulphate, earlier sowing, and/or seed blanching and soaking in water, have been demonstrated to reduce the potential concern of using red vetchling for animal feeds due to the ODAP content [9,29]. Indeed, although not analyzed, the ODAP content in diets including red vetchling meal used here seemed not to induce neurotoxicity as no abnormal swimming behavior was observed in rainbow trout juveniles fed the different experimental diets, regardless of the % of inclusion of red vetchling (up to 30% of the DM; T100). Indeed, the present results are in line with the lower sensitivity to ODAP reported in fish species than in terrestrial animals [30], and reports of the safe inclusion of red vetchling at 30–70% in the diets for poultry, pigs, and sheep [11]. On the other hand, several ANFs other than ODAP have been found in red vetchling, including those commonly present in grain legumes (tannins, phytic acid, oligosaccharides, protease inhibitors (trypsin and chymotrypsin inhibitors), amylase inhibitors, and lectins; reviewed in [11]).

Phytic acid acts as a chelating agent of other minerals besides phosphorus (e.g., Ca, Mg, Zn, Fe), thereby reducing the activity of digestive enzymes and subsequent animal performance [31,32]. However, a lower content of phytate in red vetchling meal (1.69 ± 0.28 mg/g) than in SBM, green pea (*Pisum sativum*), and Narbonne vetch (*Vicia narbonensis*) meals (2.26 to 4.90 mg/g) was observed [8]. In contrast, a high presence of trypsin and chymotrypsin inhibitors was evidenced in red vetchling (with 15.14–16.28 and 16.60–20.78 trypsin and chymotrypsin inhibitor units per mg, respectively) compared to that of other legumes [12]. The presence of these proteinaceous ANFs might partially explain the lower apparent digestibility coefficient (ADC) of protein in feeds containing the highest level of red vetchling meal (30% of DM, T100). A reduced protein ADC in *Labeo rohita* was also observed when fed for 60 days with a diet containing a similar level (34%) of another closely related species, red chickling vetch (*Lathyrus sativus*; [33]). Furthermore, the lower protein ADC of the T100 diet is consistent with lower growth, weight gain, specific growth rate, feed conversion rate, and condition factor registered in fish fed this diet. Barse et al. [33] did not report lower values for these parameters using the diet with the high content (34%) of red chickling vetch, probably because the evaluated feeding period was shorter (60 days). Also, no difference in protein ADC was reported in rainbow trout or Nile tilapia (*Oreochromis niloticus*) fed diets containing similar levels of red chickling vetch (30% of DM) after a 12-day period when compared to the corresponding reference diet [34]. Different processing techniques (e.g., fermentation, extrusion, germination, and autoclaving) were reported to increase the nutritional value of red chickling vetch [35,36], and might be explored to achieve a better implementation of red vetchling meal in aquafeeds.

Polysaccharides are another important ANF in red vetchling to be considered, and particularly NSPs. The inclusion of different types and levels of polysaccharides have shown different effects on diet digestibility parameters, with some classes of polysaccharide having greater effects than others [37]. Legume seeds contain a wide range of oligosaccharides that are not hydrolyzed by the endogenous enzymes from monogastrics, altering nutrient digestion [5] and, in particular, are known to contain specific NSPs decreasing intestinal transit, gastric emptying, and/or glucose absorption [12,13,38,39]. Reduced growth performance and the activity of some digestive enzymes (e.g., trypsin) have been reported in rainbow trout fed diets containing specific NSPs and/or plant protein sources containing high levels of different NSPs [39,40,41,42]. Therefore, the presence of some particular NSPs in red vetchling might also be responsible for the lower protein apparent digestibility of the T100 diet.

The profile of NSPs in red vetchling was similar to that of Narbonne vetch, another promising legume seed locally produced in Europe, with arabinose and glucose being the most abundant, and rhamnose and fucose not being detected [39]. Several reports evidenced how, as a consequence of NSP availability in legume seeds, substrates’ accessibility to enzymes (and activity) and carbohydrates absorption were reduced, particularly glucose and plasma glucose levels (reviewed in [14]). Although in the present work, no enzyme activity was evaluated, previous studies have demonstrated that *Lathyrus spp*. contain amylase inhibitors [43]. Furthermore, the reduced enzyme activity of amylase and its gene expression, as well as the altered metabolism of carbohydrates at a transcriptional level reported in rainbow trout fed diets containing similar levels of Narbonne vetch [39], might also occur when they are fed diets containing red vetchling as they contain a similar profile of NSPs. In fact, this reported effect of NSPs on digestive enzymes is consistent with lower HSI, lower hepatocyte surface coverage, and decreased post-prandial plasma glucose and triglyceride levels in fish fed diets containing increasing levels of red vetchling.

Lower glucose assimilation in the intestine would induce lower plasma glucose levels and, therefore, lower accumulation of carbohydrates in liver, reflected by lower HSI and reduced hepatocyte surface coverage (less hepatocyte vacuolization). Although in Toledo-Solís et al. [39], lower amylase activity and gene expression, as well as altered carbohydrates metabolism, were correlated with lower hepatocyte surface coverage, no differences in plasma glucose 24 h post-feeding were observed when rainbow trout were fed a diet in which 66% SBM was replaced with Narbonne vetch. In the present study, a reduced glucose plasma level was clearly observed at 3 and 6 h post-feeding and remained even at 24 h post-feeding in fish fed T66 and T100 diets (66 and 100% of SBM replacement by red vetchling). Those differences between both legume seeds might be related to the abundance of each NSP in the soluble fraction. The soluble form of NSPs has been shown to have a major impact on growth and digestive enzymes in rainbow trout [41], being values for arabinose, xylose, and galacturonic acid in soluble form more than double in red vetchling than in Narbonne vetch.

Altered plasma triglyceride content has been previously associated with impaired glucose metabolism [39]. Present lower post-prandial plasma levels of triglycerides in rainbow trout 3 and 6 h after being fed diets containing red vetchling might also be due to their content in NSPs. Diets containing specific NSPs or plant protein sources rich in NSPs are known to decrease fat digestibility (lipase activity) and impair lipid absorption and accumulation since bile salts are entrapped and/or their hydrolysis increased [13,14,44]. Indeed, the fatty acid profile of red vetchling meal has not been evaluated here, and future studies should be conducted to explore whether the fatty acid profile of red vetchling might affect the lipid profile in fish fillet.

Finally, red vetchling did not induce major histopatho-morphological alterations of the liver or proximal intestine. Dietary inclusion up to 30% only led to the increased density of pyknotic nuclei in hepatocytes and a wider muscular layer of the proximal intestine. The cause of these effects seems to be unclear. A prolonged consumption of soluble NSPs was associated with an increased size and length of the digestive organs in African catfish (*Clarias gariepinus*; [40]). NSPs, in particular soluble pectin, induced adipose degeneration and fibrosis in the liver of yellow catfish (*Pelteobagrus fulvidraco*) but only when included at a high level (30%; [45]). Independently of the causes, compared with 30% inclusion of Narbonne vetch (leading to a loss of brush border integrity, a high level of villi fusion, reduced goblet cell density, and reduced width of submucosa, muscular and serosa layers; [20]), 30% inclusion of red vetchling (100% SBM replacement) did not have such a large impact on the intestine. Therefore, 66% SBM replacement (20% inclusion) of red vetchling seemed to be a much safer alternative for European aquaculture, even when compared to that of Narbonne vetch previously treated with exogenous enzymes [39]. Moreover, it might also be a cost-effective alternative for SBM. While current prices of SBM are around USD 450–600 per tn (https://www.cmegroup.com/; accessed on 1 September 2023) depending on the country of origin and season, while prices for red vetchling on the Spanish market are around USD 200–300 per tn (local producer pers. communication).

## 5. Conclusions

The use of red vetchling as a locally produced crop for reducing European dependency on SBM imports from third countries for rainbow trout diets was investigated here. Successful SBM replacement of up to 66% can be achieved without any negative effects on fish growth performance and proximal intestine morpho-histopathology. Furthermore, although 66% of SBM replacement with red vetchling altered liver fat deposit and blood biochemistry, this does not seem to greatly compromise fish physiology. Considering protein apparent digestibility and carbohydrate content, particularly that of NSPs, improved use of red vetchling might be expected when applying commercially available exogenous enzymes such as cellulases and pectinases, and/or using red vetchling cultivars with a low content in these compounds. Moreover, the presence of ODAP did not induce any neurotoxic effect in rainbow trout, at least when 30% dietary inclusion of red vetchling was tested. Due to red vetchling adaptability to a diverse set of environments, including poor and dry soils, meal from the seeds of this legume certainly represents a very promising alternative material to reduce SBM imports, allowing wise exploitation of southern Europe croplands, and reducing the carbon footprint of European aquafeeds. Furthermore, its inclusion in aquafeeds might help to increase the profitability of intercropping (polycropping) approaches, with better performances than the established monocropping [9,46].

## Figures and Tables

**Table 1:** Ingredients of formulation and proximate composition of experimental diets.

Ingredients (g/100 g)	Diets					
	Control	T8	T16	T33	T66	T100
Fishmeal LT	25.00	25.00	25.00	25.00	25.00	25.00
Red vetchling	0.00	2.50	5.00	10.00	20.00	30.00
Soybean meal	30.00	27.50	25.00	20.00	10.00	0.00
Wheat gluten	12.02	13.02	14.01	16.01	19.99	23.98
Wheat meal	13.46	12.37	11.27	9.27	5.13	0.99
Fish oil	6.75	6.75	6.75	6.75	6.75	6.75
Vegetable oil	6.75	6.75	6.75	6.75	6.75	6.75
Soy lecithin	1.45	1.43	1.40	1.35	1.25	1.14
Premix ^a^	2.00	2.00	2.00	2.00	2.00	2.00
Binder ^b^	2.00	2.00	2.00	2.00	2.00	2.00
Methionine	0.52	0.54	0.54	0.56	0.56	0.56
Lysine	0.05	0.12	0.18	0.31	0.57	0.83
Total	100.00	100.00	100.00	100.00	100.00	100.00
**Proximate composition** (% on dry matter)						
Moisture	5.56 ± 0.33	3.98 ± 0.15	4.10 ± 0.25	4.79 ± 0.35	4.93 ± 0.30	5.27 ± 0.11
Crude protein	42.67 ± 0.92	42.34 ± 0.38	42.34 ± 0.47	42.61 ± 1.60	42.43 ± 1.26	42.78 ± 0.63
Crude fat	20.82 ± 0.18	22.01 ± 0.33	21.06 ± 0.71	20.86 ± 0.20	20.85 ± 0.16	20.21 ± 0.31
Crude fiber	1.91 ± 0.16	1.81 ± 0.20	1.69 ± 0.15	1.98 ± 0.14	1.91 ± 0.00	1.91 ± 0.18
Ash	8.17 ± 0.05	7.63 ± 0.25	8.46 ± 0.17	7.30 ± 1.05	7.12 ± 0.29	8.37 ± 0.25

^a^ Vitamin and mineral premix TECNOVIT; ^b^ Guar gum; Values are expressed as mean ± standard deviation.

**Table 2:** Proximate composition of red vetchling (*L. cicera*) seeds.

Proximate Composition (% on Dry Matter)	
Moisture	3.62 ± 0.22
Crude protein	23.82 ± 0.47
Crude fat	2.03 ± 0.06
Ash	3.78 ± 0.07
Nfe *	70.37 ± 0.39
**Amino acid content** (g/100 g ingredient)	
Essential amino acid	
Arginine	1.88 ± 0.12
Histidine	0.38 ± 0.02
Isoleucine	0.72 ± 0.12
Leucine	1.45 ± 0.09
Lysine	1.75 ± 0.10
Phenylalanine	0.88 ± 0.04
Valine	1.07 ± 0.11
Methionine	0.24 ± 0.01
Threonine	0.91 ± 0.05
Non-essential amino acid	
Alanine	1.21 ± 0.05
Aspartic acid	2.75 ± 0.06
Glutamic acid	4.19 ± 0.12
Glycine	0.97 ± 0.04
Serine	1.26 ± 0.02
Tyrosine	0.71 ± 0.02
Cysteine	0.15 ± 0.01
Proline	1.89 ± 0.20

* Nitrogen-free extract. Values are expressed as mean ± standard deviation.

**Table 3:** Monomeric composition of non-starch polysaccharides (NSP; g/100 g ingredient) content of red vetchling (*L. cicera*) seeds.

NSPs (mg/g Ingredient)	Soluble	Insoluble	Total
Rhamnose	nd	nd	nd
Fucose	nd	nd	nd
Arabinose	0.84 ± 0.18	1.98 ± 0.18	2.79 ± 0.33
Xylose	0.31 ± 0.13	0.68 ± 0.12	1.03 ± 0.17
Mannose	0.26 ± 0.05	nd	0.26 ± 0.05
Galactose	0.14 ± 0.04	0.24 ± 0.05	0.38 ± 0.05
Glucose	0.79 ± 0.18	3.15 ± 0.32	3.82 ± 0.36
Galacturonic acid	0.46 ± 0.22	1.71 ± 0.20	2.28 ± 0.46
**Total**	**2.78 ± 0.46**	**7.68 ± 0.66**	**10.40 ± 0.84**

*nd*, not detected. Values are expressed as mean ± standard deviation.

**Table 4:** Amino acid profile of experimental diets.

Amino Acid Content (g/100 g Ingredient)	Diets					
	Control	T8	T16	T33	T66	T100
Essential amino acid						
Arginine	2.10 ± 0.01	2.12 ± 0.05	1.97 ± 0.01	1.99 ± 0.01	1.92 ± 0.01	1.87 ± 0.02
Histidine	1.03 ± 0.38	1.09 ± 0.44	1.52 ± 0.32	1.21 ± 0.08	1.31 ± 0.08	1.27 ± 0.09
Isoleucine	1.77 ± 0.01	1.80 ± 0.10	1.65 ± 0.03	1.62 ± 0.06	1.65 ± 0.01	1.63 ± 0.13
Leucine	2.89 ± 0.07 ^a^	2.92 ± 0.03 ^a^	2.63 ± 0.05 ^b^	2.63 ± 0.07 ^b^	2.64 ± 0.00 ^b^	2.73 ± 0.05 ^ab^
Lysine	2.81 ± 0.06	2.84 ± 0.02	2.87 ± 0.12	2.73 ± 0.11	2.87 ± 0.05	2.95 ± 0.08
Phenylalanine	1.96 ± 0.15	1.95 ± 0.20	1.98 ± 0.10	1.93 ± 0.02	1.96 ± 0.04	2.00 ± 0.02
Valine	2.42 ± 0.04	2.36 ± 0.05	2.19 ± 0.10	2.20 ± 0.09	2.24 ± 0.05	2.21 ± 0.13
Methionine	1.13 ± 0.10	1.16 ± 0.10	1.03 ± 0.11	1.08 ± 0.04	1.17 ± 0.09	1.12 ± 0.03
Threonine	1.28 ± 0.11	1.35 ± 0.12	1.13 ± 0.07	1.14 ± 0.12	1.11 ± 0.05	1.24 ± 0.12
Non-essential amino acid						
Alanine	2.07 ± 0.01 ^a^	2.07 ± 0.00 ^a^	1.93 ± 0.11 ^ab^	1.93 ± 0.03 ^ab^	1.88 ± 0.07 ^ab^	1.86 ± 0.02 ^b^
Aspartic acid	3.40 ± 0.06 ^a^	3.37 ± 0.03 ^a^	2.96 ± 0.11 ^b^	2.93 ± 0.06 ^b^	2.78 ± 0.00 ^bc^	2.69 ± 0.04 ^c^
Glutamic acid	8.21 ± 0.11 ^bc^	8.25 ± 0.08 ^bc^	7.65 ± 0.25 ^c^	8.07 ± 0.22 ^bc^	8.54 ± 0.09 ^b^	9.20 ± 0.08 ^a^
Glycine	1.91 ± 0.04 ^ab^	1.90 ± 0.01 ^ab^	1.95 ± 0.10 ^ab^	2.02 ± 0.06 ^a^	1.98 ± 0.02 ^ab^	1.77 ± 0.05 ^b^
Serine	1.38 ± 0.31	1.57 ± 0.31	1.26 ± 0.28	1.28 ± 0.36	1.23 ± 0.10	1.59 ± 0.29
Tyrosine	1.02 ± 0.03	1.12 ± 0.01	1.12 ± 0.19	1.10 ± 0.09	1.08 ± 0.02	1.16 ± 0.06
Cysteine	0.34 ± 0.02	0.35 ± 0.02	0.32 ± 0.01	0.33 ± 0.00	0.34 ± 0.00	0.35 ± 0.00
Proline	1.08 ± 0.01	1.13 ± 0.02	1.07 ± 0.00	1.19 ± 0.05	1.17 ± 0.04	1.26 ± 0.03

Values are expressed as mean ± standard deviation. Different superscript letters within each row denote significant differences among experimental groups (one-way ANOVA, *p* < 0.05; *n* = 3).

**Table 5:** Growth performance and protein apparent digestibility in rainbow trout fed with experimental diets.

Day	Parameter	Diets					
		Control	T8	T16	T33	T66	T100
0	IBW (g)	10.36 ± 0.16	10.25 ± 0.05	10.37 ± 0.08	10.34 ± 0.04	10.37 ± 0.13	10.34 ± 0.05
	IFL (cm)	9.49 ± 0.08	9.44 ± 0.07	9.47 ± 0.05	9.45 ± 0.04	9.4 ± 0.06	9.45 ± 0.03
90	FBW (g)	150.66 ± 2.27 ^a^	144.90 ± 13.20 ^a^	146.05 ± 8.28 ^a^	142.14 ± 3.60 ^a^	140.38 ± 8.74 ^a^	100.52 ± 5.78 ^b^
	FFL (cm)	22.69 ± 0.16 ^a^	22.39 ± 0.62 ^a^	22.34 ± 0.42 ^a^	22.03 ± 0.14 ^a^	22.11 ± 0.28 ^a^	20.15 ± 0.35 ^b^
	WG (%)	1357.79 ± 18.21 ^a^	1313.70 ± 136.29 ^a^	1308.58 ± 87.12 ^a^	1285.39 ± 36.59 ^a^	1254.58 ± 97.69 ^a^	871.87 ± 60.07 ^b^
	SGR (%/day)	2.98 ± 0.01 ^a^	2.94 ± 0.11 ^a^	2.94 ± 0.07 ^a^	2.91 ± 0.04 ^a^	2.89 ± 0.08 ^a^	2.53 ± 0.07 ^b^
	FCR	0.79 ± 0.01 ^a^	0.80 ± 0.03 ^a^	0.80 ± 0.02 ^a^	0.81 ± 0.01 ^a^	0.83 ± 0.03 ^a^	1.00 ± 0.04 ^b^
	CF	1.29 ± 0.02 ^a^	1.29 ± 0.02 ^a^	1.31 ± 0.01 ^a^	1.33 ± 0.01 ^a^	1.30 ± 0.03 ^a^	1.23 ± 0.01 ^b^
	HSI (%)	1.42 ± 0.12 ^a^	1.35 ± 0.04 ^ab^	1.30 ± 0.08 ^ab^	1.20 ± 0.10 ^ab^	1.13 ± 0.02 ^b^	1.29 ± 0.09 ^ab^
	VSI (%)	12.00 ± 0.90	11.81 ± 0.58	11.16 ± 0.28	10.87 ± 0.25	10.19 ± 2.62	11.69 ± 1.52
**Apparent digestibility of the protein and proximate composition of the fish fillet (%)**							
ADCprotein ^1^		92.89 ± 0.18 ^b^	91.66 ± 0.18 ^b^	94.38 ± 0.52 ^a^	92.44 ± 0.47 ^b^	93.88 ± 0.38 ^a^	90.03 ± 0.84 ^c^
Moisture		1.74 ± 0.15 ^ab^	1.18 ± 0.13 ^b^	1.04 ± 0.06 ^b^	2.41 ± 0.88 ^a^	1.74 ± 0.35 ^ab^	0.68 ± 0.28 ^b^
Crude protein		15.56 ± 0.21 ^a^	16.31 ± 0.54 ^a^	13.06 ± 0.04 ^b^	12.5 ± 0.50 ^b^	11.44 ± 0.19 ^c^	11.06 ± 0.31 ^c^
Ash		4.69 ± 0.06 ^a^	4.48 ± 0.05 ^a^	4.62 ± 0.38 ^a^	4.58 ± 0.43 ^a^	3.25 ± 0.15 ^b^	2.66 ± 0.15 ^b^

^1^ Apparent Digestibility coefficient of the protein; *IBW*, initial body weight in g; *IFL*, initial furcal length in cm; *FBW*, final body weight in g; *FFL*, final furcal length in cm; *WG*, weight gain in %; *SGR*, specific growth rate in %/day; *FCR*, feed conversion ratio; *CF*, condition factor; *HSI*, hepato-somatic index in %; *VSI*, viscero-somatic index in %. Values are expressed as mean ± standard deviation. Different superscript letters within each row denote significant differences among experimental groups (one-way ANOVA, *p* < 0.05; *n* = 3).

**Table 6:** Amino acid profile of fish fillet from rainbow trout fed with experimental diets.

Amino Acid Content (g/100 g Dry Ingredient)	Diets					
	Control	T8	T16	T33	T66	T100
Essential amino acid						
Arginine	3.33 ± 0.24	3.73 ± 0.05	3.83 ± 0.14	3.80 ± 0.31	3.86 ± 0.34	4.06 ± 0.44
Histidine	2.90 ± 0.33	2.79 ± 0.47	2.81 ± 0.49	2.86 ± 0.40	3.05 ± 0.50	2.13 ± 0.72
Isoleucine	4.31 ± 0.32	4.38 ± 0.09	4.58 ± 0.16	4.32 ± 0.32	4.37 ± 0.13	4.68 ± 0.42
Leucine	7.62 ± 0.44	7.24 ± 0.34	7.51 ± 0.67	7.22 ± 0.37	7.69 ± 0.36	7.69 ± 0.57
Lysine	6.82 ± 0.59	6.91 ± 0.76	7.08 ± 0.31	6.96 ± 0.45	7.12 ± 0.81	8.08 ± 0.64
Phenylalanine	3.55 ± 0.13	3.56 ± 0.34	3.70 ± 0.14	3.53 ± 0.19	3.69 ± 0.30	4.12 ± 0.50
Valine	3.55 ± 0.18	3.36 ± 0.15	3.52 ± 0.33	3.31 ± 0.17	3.53 ± 0.15	3.53 ± 0.28
Methionine	2.72 ± 0.14	2.75 ± 0.36	2.86 ± 0.09	2.63 ± 0.19	2.76 ± 0.30	3.23 ± 0.54
Threonine	2.28 ± 0.17	2.16 ± 0.04	2.26 ± 0.22	2.31 ± 0.16	2.50 ± 0.22	2.41 ± 0.33
Non-essential amino acid						
Alanine	3.52 ± 0.11	3.38 ± 0.42	3.46 ± 0.26	3.37 ± 0.20	3.47 ± 0.37	3.09 ± 0.12
Aspartic acid	6.60 ± 1.18	6.67 ± 0.78	6.65 ± 0.21	7.16 ± 0.46	7.50 ± 0.61	7.06 ± 0.47
Glutamic acid	9.55 ± 0.72	9.84 ± 0.54	9.97 ± 0.17	10.27 ± 0.74	10.47 ± 0.51	10.51 ± 0.69
Glycine	2.91 ± 0.28	2.95 ± 0.26	3.02 ± 0.12	3.06 ± 0.23	3.17 ± 0.29	2.89 ± 0.01
Serine	4.21 ± 0.80	3.81 ± 0.32	3.96 ± 0.64	4.08 ± 0.31	4.33 ± 0.46	3.73 ± 0.30
Tyrosine	1.75 ± 0.63 ^b^	2.36 ± 0.40 ^ab^	2.49 ± 0.02 ^ab^	2.05 ± 0.58 ^ab^	1.83 ± 0.85 ^b^	2.89 ± 0.24 ^a^
Cysteine	0.21 ± 0.03	0.22 ± 0.02	0.26 ± 0.00	0.28 ± 0.09	0.28 ± 0.10	0.36 ± 0.11
Proline	2.22 ± 0.10	2.06 ± 0.02	2.15 ± 0.29	2.00 ± 0.19	2.05 ± 0.11	2.01 ± 0.13

Values are expressed as mean ± standard deviation. Different superscript letters within each row denote significant differences among experimental groups (one-way ANOVA, *p* < 0.05; *n* = 3).

**Table 7:** Histopathology analysis in the proximal intestine of rainbow trout at 90 days of feeding with the experimental diets.

Parameter	Diets					
	Control	T8	T16	T33	T66	T100
Width of submucosa layer (µm)	8.82 ± 0.72	8.17 ± 0.54	8.90 ± 0.66	7.93 ± 0.81	6.99 ± 1.50	6.06 ± 1.86
Width of serosa layer (µm)	13.44 ± 2.62	16.54 ± 1.79	15.83 ± 3.28	15.66 ± 0.63	13.46 ± 0.65	12.47 ± 1.80
Height of villi (µm)	343.63 ± 69.91	357.68 ± 35.12	354.80 ± 15.05	338.31± 27.69	341.42 ± 28.35	339.13 ± 16.04
Height of enterocytes (µm)	14.46 ± 1.49	15.47 ± 0.26	15.05 ± 0.74	15.23 ± 0.38	15.20 ± 1.65	14.54 ± 0.94
Density of goblet cells (cells/mm)	59.11 ± 5.32	58.20 ± 7.30	61.82 ± 18.04	56.79 ± 8.56	50.33 ± 5.75	61.61 ± 8.30

Values are expressed as mean ± standard deviation.

**Table 8:** Postprandial analysis of plasma glucose and triglycerides from rainbow trout fed with experimental diets.

Parameter	Diet	Time Post-Feeding		
		3 h	6 h	24 h
Glucose (mg/dL)	Control	100.37 ± 10.22 ^ab^	79.90 ± 2.41 ^ab^	100.18 ± 13.18 ^a^
	T8	117.31 ± 12.14 ^a^	71.35 ± 5.23 ^bc^	93.54 ± 11.97 ^a^
	T16	90.53 ± 7.77 ^bc^	86.26 ± 4.57 ^a^	81.71 ± 2.94 ^ab^
	T33	80.47 ± 3.87 ^bc^	71.48 ± 8.70 ^bc^	81.13 ± 8.46 ^ab^
	T66	73.59 ± 9.97 ^c^	69.55 ± 6.33 ^bc^	66.91 ± 7.23 ^b^
	T100	73.82 ± 7.46 ^c^	62.45 ± 3.11 ^c^	67.82 ± 5.33 ^b^
Triglycerides (mg/dL)	Control	377.78 ± 48.02 ^a^	369.62 ± 52.25 ^a^	273.61 ± 85.61
	T8	229.72 ± 36.48 ^b^	314.78 ± 31.31 ^ab^	225.63 ± 29.77
	T16	213.31 ± 49.73 ^bc^	29255 ± 12.06 ^b^	260.10 ± 43.93
	T33	223.00 ± 20.75 ^bc^	180.26 ± 14.58 ^c^	225.63 ± 68.79
	T66	203.75 ± 48.33 ^bc^	177.30 ± 35.47 ^c^	260.10 ± 43.93
	T100	134.75 ± 24.68 ^c^	219.27 ± 12.28 ^c^	225.63 ± 68.79

Values are expressed as mean ± standard deviation. Different superscript letters within each column denote significant differences among experimental groups (one-way ANOVA, *p* < 0.05; *n* = 3).

## Data Availability

All data are available upon request.
